# Special Issue: “New Trends in Alzheimer’s Disease Research: From Molecular Mechanisms to Therapeutics: 2nd Edition”

**DOI:** 10.3390/ijms26157175

**Published:** 2025-07-25

**Authors:** Ramón Cacabelos

**Affiliations:** International Center of Neuroscience and Genomic Medicine, 15165 Bergondo, Spain; rcacabelos@euroespes.com

Over the last 30 years, the gradual increase in cases of senile dementia has led to it becoming one of the most concerning health problems in developed countries. In 2021, approximately 57 million people worldwide were living with dementia, with Alzheimer’s disease (AD) accounting for 60–70% of cases. The global prevalence of dementia (~0.7% of the population) equated to around 51.6 million in 2019, with new global dementia cases numbering about 10 million per year and the incidence of Alzheimer’s and related dementias rising sharply from 2.92 million new cases in 1990 to 7.24 million in 2019 (+148%). Alzheimer’s comprises ~5–8 new cases per 1000 person-years in older adults, with rates doubling every 5–6 years after age 65. In 2025, an estimated 7.2 million Americans (1 in 9) aged 65+ live with AD. Younger-onset cases (<65) affect approximately 200,000 Americans, while the number of people in the country who die annually due to AD is similar. The disease is the fifth-highest cause of death in those 65+ and contributes significantly to excess mortality. In the EU, the prevalence of dementia is in the range of ~5–8.7% in the elderly. China alone accounts for approximately 16 million dementia cases, nearly 30% of the total; its national dementia incidence is rising ~2.9% annually, with an expected 400 million cases in 2035 in people over 60. Japan has one of the highest prevalence rates: ~3079 cases per 100,000 population (about 3%). This is well above global averages (667 per 100 k), and Japan’s dementia-related deaths are projected to be the highest in the world by 2040. Asia is now the leading region in new dementia cases (nearly 50% of new cases annually) [1,2,3,4].

Over the past five years, the development of Alzheimer’s research projects has slowed, particularly due to the impact of the COVID-19 pandemic. However, following the pandemic, the number of studies has rebounded, with 14,209 publications in 2020, 15,837 in 2021, 16,305 in 2022, 17,080 in 2023, and 18,394 in 2024. By 2025, scientific publications on Alzheimer’s are expected to exceed 20,000.

The most significant advances in Alzheimer’s research over the past five years have focused on understanding the pathogenesis and molecular mechanisms responsible for the disease, early diagnosis and the development of antemortem biomarkers, and new treatment options.

The core proteinopathy hypotheses associated with extracellular amyloid-β (Aβ) deposits in senile plaques and intracellular hyperphosphorylated tau-related neurofibrillary tangle (NFT) formation continue to be fundamental factors in the pathogenesis of AD. The amyloid cascade hypothesis remains foundational: misprocessing of the amyloid precursor protein yields Aβ monomers that oligomerize, disrupt calcium homeostasis via ion channel formation in neuronal membranes, and drive downstream tau pathology, oxidative stress, and neuroinflammation. Recent structural and biochemical insights into soluble Aβ oligomers have refined our understanding of their role in seeding both synaptic dysfunction and tau phosphorylation [5]. Evidence continues to support the tau hypothesis, which links abnormal tau hyperphosphorylation and aggregation into NFTs with neuronal microtubule destabilization and cell death. Imaging breakthroughs now allow in vivo tau and Aβ PET visualization, aiding early diagnosis and monitoring response to anti-Aβ and anti-tau drugs [6].

Neuroinflammation and immune dysregulation are present in brains with AD. NLRP3 inflammasome activation by Aβ and hyperphosphorylated tau leads to chronic microglial-driven inflammation, lysosomal damage, and defective Aβ clearance. The failure of inflammasome-mediated phagocytosis contributes to a vicious cycle of neurotoxicity. These findings have catalyzed the development of NLRP3 inhibitors and other microglia-targeted immunomodulators under preclinical review [7,8,9,10].

Lipid metabolism dysregulation, alterations in blood–brain barrier (BBB) integrity, and ion flux changes also contribute to the pathogenesis of AD. A novel hypothesis argues that BBB dysfunction permits the infiltration of lipids (LDL, free fatty acids), initiating neuronal damage and Aβ/tau pathologies. This extends traditional cholesterol hypotheses [11,12,13].

Microbiome-derived bile acids, most notably deoxycholic acid, have been shown to bind gamma-secretase components (nicastrin), increasing Aβ production. This suggests that the gut–brain axis modulates amyloidosis [14,15].

According to the ion channel hypothesis, soluble Aβ oligomers are found to form cationic pores in neuronal membranes, causing calcium influx, mitochondrial dysfunction, oxidative stress, and apoptosis [16].

Elevated oxidative stress and DNA double-strand break (DSB) accumulation have been documented in brains with early-stage AD, particularly near Aβ plaques, coinciding with reduced DNA-PK levels and repair capacity. Accumulation of γH2AX and COMET-assessed DSBs in the hippocampus/cortex adjacent to Aβ plaques and decreased DNA-PKcs expression link oxidative stress to repair deficits [17].

Aβ-induced ROS and DSB accumulation via oxidative stress highlights how reduced DNA-PK repair worsens neuronal health and contributes to degeneration [18]. Aβ exposure elevates ROS, suppresses DNA-PK activity, and impairs NHEJ repair, triggering neuronal apoptosis [19]. Postmortem hippocampal data show that DNA-PKcs downregulation correlates with γH2AX foci and cognitive decline in MCI/AD cases. Oxidative stress driven by Aβ triggers elevated ROS, which produce DNA DSBs (γH2AX-positive) in neurons and glia. DNA-PKcs, a key enzyme in non-homologous end joining (NHEJ), is significantly downregulated in early AD, compromising DNA repair. The resulting genomic instability contributes to neuronal dysfunction, synaptic loss, and cell death, amplifying AD progression [20].

Advances in the genomics of AD (>1500 genes associated with AD-related neurodegeneration) have highlighted several issues, which are outlined below. (i) Polygenic risk scores and cross-ancestry GWAS: A European-derived polygenic risk score (PGS_AD) was tested in 17 countries and multiple ancestries, independently predicting AD risk and age of onset—even after adjusting for APOE—though performance declined in African and East Asian populations, highlighting both transferability and limitations across ancestries [21]. A deep learning model using whole-genome sequencing (WGS) data achieved improved predictive performance, demonstrating the integration of local and global genomic annotations for AD risk classification [22]. (ii) Whole-genome and multi-ancestry sequencing studies: Compiling >400,000 structural variants (SVs), a study linked numerous SVs to AD risk, underscoring the importance of SVs in unexplained heritability [23]. Multi-ancestry WGS (*n* ≈ 49,000) identified 16 novel AD-associated loci tied to neuronal connectivity, lipid metabolism, and immune signaling (43% non-European participants), emphasizing genetic diversity in risk assessment. (iii) Diverse ancestry and population-specific genetics: In a Korean cohort, a novel locus (APCDD1), rare cis-regulatory variants in excitatory neurons, STR expansions, and CNVs were uncovered, highlighting population-specific contributions to AD risk. (iv) Polygenic risk and functional genomics integration: Pathway-specific and composite genetic risk scores offer improved predictive power and new insights into AD pathogenesis, including immune and lipid pathways. A study connected AD polygenic risk to T cell gene expression, uncovering immune-related gene networks influenced by AD genetics. (v) Population diversity and data sharing infrastructures: Another study demonstrated four features of comprehensive genetic data across diverse populations, serving as a vital resource for future genomic discovery [24,25,26,27,28].

Future directions in AD genomics research include broader population sequencing to reduce bias and uncover novel genetic variants across global ancestors; multi-omics integration, overlaying genomics with transcriptomics, proteomics, and immune profiling for mechanistic insights; clinical genomics translation, translating predictive genomics into pre-symptomatic screening and personalized intervention strategies; and functional validation, interrogating novel variants (e.g., SVs, regulatory loci) to establish causality.

Concerning epigenetic and transcriptomic alterations, global DNA hypomethylation and promoter-specific hypermethylation in genes like BDNF, NEP, APP, and BACE1-AS have been found in AD brain tissue [29]. Elevated HDAC2 expression and corresponding H3 hypoacetylation correlate with impaired synaptic plasticity and memory. HDAC inhibitors (e.g., sodium butyrate, vorinostat) show memory-enhancing effects in mouse models [30]. Dysregulated microRNAs (e.g., miR-9, miR-128, miR-15a) are implicated in synaptic decline and neuroinflammatory gene expression profiles. Profiling of human cortical samples found reduced miR-9/128 and increased miR-15a, correlating with the downregulation of synaptic genes and upregulation of inflammatory markers in microglia [31]. Single-cell RNA-seq studies have revealed cell-type-specific gene regulatory network changes, especially in hippocampal neurons and glial cells. When applied to ~95,000 hippocampal nuclei from AD and control brains, this method uncovered cell-specific regulatory modules linked to inflammation, lipid metabolism, and synaptic function in neurons and glia [32]. Built upon scRNA-seq and spatial data, an atlas identified subpopulation-specific transcriptomic signatures across cell types, including astrocytic resilience programs and microglial activation states [33].

Multi-omics integration is now being used to map early-stage AD triggers like metabolic disturbances, lipid signaling, and neurovascular unit dysfunction. Integrated analysis (epigenome, transcriptome, proteome, and metabolome) has revealed AD-associated regulatory modules and metabolic dysfunction [34]. One study combined metabolomic and transcriptomic analyses to identify immune, glial, synaptic, and metabolic disturbances in hippocampi with AD [35]. The integration of diverse omics layers may be useful to identify targets in AD and facilitate precision medicine [36].

Gut microbiota-derived metabolites (e.g., bile acids) influence gamma-secretase activity, linking peripheral dysbiosis to central amyloidogenesis [37]. Mouse AD models exhibit gut dysbiosis and elevated bile acids, linking microbial metabolites to brain pathology and cognitive decline [38]. Combining microbiome, metabolome, and MRI data in humans reveals peripheral metabolite–brain structure–cognition associations, supporting gut–brain mechanistic links [39]. Secondary bile acids modulate gut microbiota composition, intestinal barrier function, inflammation, and neurodegenerative processes [40].

With regard to novel therapeutic candidates, there are several options. Experimental enzyme and protein modulators include BACE1 inhibitors, gamma-secretase modulators, selective Aβ-tau dual inhibitors, PPI modulators, and PROTACs, aiming to degrade pathogenic proteins [41,42,43,44]. Sirtuin activators (e.g., SIRT1 enhancers) promote neuroprotection via deacetylation, reduce tau aggregates, and enhance mitochondrial resilience. Some studies demonstrate the neuroprotective effects of boosting SIRT1 activity, such as the deacetylation of tau, reduced aggregates, and preserved mitochondrial integrity in AD mice [45]. Some other strategies using different epigenetic drugs have been proposed. HDAC and DNMT inhibitors, along with NSAIDs, are under preclinical evaluation again to reduce neurodegeneration and improve synaptic health via epigenetic pathways [46] and new modalities of pleiotropic epigenetic compounds have been successfully tested in transgenic AD models [47,48]. NLRP3 inflammasome inhibitors (OLT1177) are in development to break the inflammatory feedback loop hindering Aβ clearance [49,50].

Advanced in vitro/in vivo models show that single-cell and spatial transcriptomics, organoids, and humanized animal models with BBB features are enabling deeper insight into cell-specific pathomechanisms [51]. New computational platforms (e.g., ScAtt) yield more accurate gene network inference, unveiling therapeutic targets in hippocampal neuron–glia circuits [32,52].

In summary, there is now a tendency towards an integrated pathogenetic paradigm, which is outlined in more detail below. (i) Genetic/amyloid initiation with Aβ oligomers and tau misfolding: Genomic studies link familial and sporadic AD to APP, PSEN1/2, and APOE4 variants, which are key drivers of Aβ overproduction and oligomerization. Misfolded Aβ seeds tau aggregation and synaptic loss [53]. Extracellular Aβ oligomers interact with tau to promote misfolding and spread in neuronal networks [54]. (ii) BBB breakdown and lipid ingress potentiate neuroinflammation and amyloidosis: BBB disruption permits LDL and fatty acids to infiltrate the brain, triggering neuroinflammation and encouraging Aβ aggregation [55]. Lipid imbalances driven by ApoE4 and diet compromise BBB integrity, exacerbating amyloidosis and inflammation. (iii) Oxidative stress and DNA damage induce genomic instability and neuronal loss: ROS and γH2AX markers in hippocampal neurons near Aβ plaques, in addition to reduced DNA-PK activity, lead to DNA damage and neuronal death. Postmortem examination of brains with AD confirms that the loss of DNA-PKcs corresponds to accumulated DNA damage and cognitive impairment. (iv) Neuroimmune dysregulation (NLRP3 activation) causes sustained inflammation: NLRP3 activation in microglia drives tau aggregation and neuronal toxicity, and the inhibition of inflammasome activity reduces IL-1β release, enhances Aβ clearance, and restores cognition. (v) Epigenetic reprogramming generates altered gene expression in synaptic and survival pathways: Global DNA hypomethylation and hypermethylated promoters (e.g., BDNF, APP) in brains with AD contribute to synaptic gene suppression. HDAC2-driven epigenetic repression of plasticity genes is reversed using HDAC inhibitors, restoring memory and synaptic function. (vi) Systemic–metabolic signaling (gut–liver–brain axis) amplifies amyloid production: Deoxycholic acid binding to γ-secretase subunits enhances Aβ generation, a direct link between gut metabolites and amyloidosis. Specific microbial metabolite elevations are associated with cognitive decline and Aβ accumulation in AD mouse models. This multifactorial model supports multi-target interventions, combining amyloid/tau reduction, lipid management, DNA repair enhancement, immunomodulation, epigenetic therapy, and microbiome/metabolic regulation to achieve comprehensive disease modification [56,57]. This model is fundamentally based on the amyloid–tau hypothesis; however, a growing number of studies question this model and cast doubt on the potential therapeutic efficacy of new treatments based on the amyloid hypothesis. Some authors propose that amyloid plaques and tau tangles are downstream byproducts, with metabolic, vascular, and mitochondrial dysfunction serving as more plausible primary triggers [58]. Others question whether amyloid reduction via monoclonal antibodies truly correlates with clinical benefit and raise concerns about potential unblinding and lack of tau-load effects [59], and others argue that amyloid hypothesis has become overly pervasive—too flexible to be falsifiable—and that Alzheimer’s likely involves a complex, multi-pathway pathology [60,61,62].

A topic of growing interest in Alzheimer’s disease is the differential geno-phenotypic profiles of men and women [63]. Some authors explored the interplay of sex chromosomes and hormones on inflammation, metabolism, and autophagy in sex-specific AD pathogenesis [64]. Others, focusing on autosomal-dominant AD and Down syndrome AD, highlight stronger neurodegeneration in females despite similar cognitive performance, and sex–gene interactions (e.g., APOE4) [65]. Transcriptomic data revealed sex-specific gene expression, pathway dysregulation, and altered cell–cell communication in glial and neuronal cells [66]. Distinct microglial metabolic and inflammatory responses can also be divided by sex, with female microglia showing heightened reactivity to amyloid and tau pathology [67]. CSF biomarkers (sTREM2, YKL-40, GFAP) indicate that in women, glial reactivity contributes more strongly to amyloid burden, tau accumulation, and hippocampal atrophy [68]. Some studies show sex-specific cognitive decline patterns, linking menopause-related estrogen loss with faster disease progression in females [69]. Women with early menopause (<48 yrs) have a higher risk of vascular-related cognitive decline and dementia compared to men [70].

In summary, women are nearly twice as likely to develop AD and show more rapid hippocampal atrophy and greater amyloid–tau pathology. Estrogen loss during menopause correlates with brain energy deficits, synaptic vulnerability, and increased amyloid accumulation. Female microglia and astrocytes exhibit more robust inflammatory reactions to amyloid/tau, influencing glial biomarker profiles and neurodegeneration. APOE4 and autosomal AD mutations display sex-specific penetrance and biomarker trajectories; women may have less cognitive reserve despite similar pathology. Earlier menopause aligns with increased vascular risk and accelerated cognitive decline in women, reinforcing the hormone–vascular–AD nexus. These insights underscore the need for sex-aware research, including sex-stratified trials and tailored interventions targeting hormonal, immune, vascular, and genetic pathways.

In the field of biomarkers, there have been many new developments in recent years, including the following: (i) Blood-based biomarkers: Phosphorylated tau (p-tau) variants (p-tau181, p-tau217, and p-tau231) reliably reflect amyloid and tau pathology, with p-tau217 showing exceptional diagnostic and prognostic performance [71,72]. Brain-derived tau (BD-tau) offers an amyloid-associated marker of active neurodegeneration, especially useful in Aβ-positive individuals [73]. Neurofilament Light Chain (NfL) reflects neurodegeneration across neurodegenerative disorders; in AD, higher plasma NfL levels predict accelerated cognitive decline [74,75]. GFAP (astrocyte activation) enhances p-tau predictive models, while emerging sensors promise point-of-care amyloid detection [76]. (ii) Digital and wearable biomarkers: Wearable devices and AI-driven analysis of gait, activity, sleep, and speech are emerging as non-invasive early AD indicators [77,78]. (iii) Multi-modal biomarker integration, combining multiple blood markers (p-tau, BD-tau, NfL, GFAP, and Aβ ratios), enhances diagnostic accuracy and staging progression in AD [79].

New treatments for AD, organized by therapeutic strategy, can be divided into the following categories: (i) Anti-Aβ immunotherapies and early intervention: Monoclonal antibodies targeting Aβ protofibrils and plaques represent the first approvals of disease-modifying therapies for AD [80,81]. Aducanumab (Aduhelm^®^) was FDA-approved on 7 June 2021, based on amyloid PET plaque reduction. Medicare limited coverage to randomized trial participants in April 2022. Clinical efficacy remains uncertain; advisory resignations followed approval, and Biogen exited the market in January 2024. Lecanemab (Leqembi^®^) received accelerated FDA approval on 6 January 2023, following the CLARITY-AD trial showing a 27% reduction in cognitive decline. It is the first agent to demonstrate consistent biomarker and clinical benefit, but was accompanied by ARIA safety concerns. The TRAILBLAZER-ALZ 2 trial showed ~35% slowed progression over 18 months, associated with ARIA (specially in APOE-4 carriers) and infusion reactions. It is specified for mild cognitive impairment or mild dementia with confirmed amyloids. These antibodies preferentially bind aggregated Aβ species—protofibrils (lecanemab) or plaques (aducanumab, donanemab)—leading to clearance and measurable biomarker changes. The clinical benefits remain modest (~27–39% slower decline); ARIA remains the key safety concern, with a major impact in patients harboring the APOE-4 allele. Ethical and economic debates persist over cost–benefit trade-offs, access, and regulatory rigor [80,81]. Emerging next-generation immunotherapies, with subcutaneous and brain-penetrating antibodies (e.g., Remternetug) are entering trials to enhance accessibility. BBB-shuttle antibodies (e.g., Trontinemab) are under development to achieve better CNS delivery. Active vaccines against tau are in Phase 1 safety trials, while passive anti-tau antibodies (e.g., Zagotenemab) are in early-stage trials. Bispecific antibodies (simultaneously targeting Aβ and inflammatory receptors) and fusion constructs enhancing effector functions are in preclinical pipelines. Antibody–drug conjugates and cell-based immunotherapy are undergoing early proof-of-concept research. Some combination strategies consider the use of monoclonals that can be combined with anti-tau agents, NLRP3 inhibitors, or metabolic drugs (e.g., GLP-1 agonists) for synergistic effects [82]. (ii) Small-molecule enzyme and protein modifiers: BACE1 and γ-Secretase modulators [83], dual-target, PROTAC, and PPI modulators [84]. (iii) Metabolic and hormonal modulators include SIRT1 activators and GLP-1 receptor agonists for cognitive preservation (clinical trials are ongoing to evaluate semaglutide and tirzepatide as neuroprotective agents in AD) [85]. (iv) Epigenetic and anti-inflammatory strategies include HDAC/DNMT inhibitors and NSAIDs [86], pleiotropic epigenetic bioproducts, and NLRP3 inflammasome inhibitors. (v) Vaccines, gene therapy, and novel biological agents include Abcelerator antibodies (e.g., trontinemab) with enhanced BBB penetration and Xanamem (Emestedastat), a 11β-HSD1 Inhibitor.

The 2025 AD drug pipeline, as reported by Cummings et al. [87], includes 138 agents in 182 clinical trials for AD treatment. A majority (74%) are disease-modifying therapies (DMTs); 17% target inflammation, 11% tau, 6% metabolism, and 2% the gut–brain axis; many engage BBB, epigenetic, and proteostasis pathways [87].

The future of AD therapy lies in combination strategies—merging immunotherapies, enzyme modulators, metabolic agents, epigenetic drugs, and BBB-targeted delivery—guided by advanced models and biomarker-driven diagnostics for tailored patient care.

A very important topic in the personalization of pharmacological AD treatment is the implantation of pharmacogenetics, whose introduction into clinical practice and into the methodology for the development of new drugs remains slow. Although the pharmacogenetics of anti-dementia drugs is relatively well known [88,89,90], its application in daily clinical practice is irregular and frankly scarce, in an extremely fragile population that consumes more than 6–10 medications daily, with the consequent risk of adverse drug reactions (ADRs) and the possibility of multiple drug–drug interactions [91].

This second edition of the Special Issue entitled **New Trends in Alzheimer’s Disease Research: From Molecular Mechanisms to Therapeutics: 2nd Edition** offers valuable contributions on different aspects of AD. Woong Jin Lee and colleagues [92] demonstrate the positive effect of mesenchymal stem cell (MSC) treatment on cerebrovascular damage in AD-transgenic animals. Juhi Shah and colleagues [93] document the effects of physical exercise and Genistein on the deleterious consequences of a high-fat, high-sugar diet on AD-related markers in mice models. Farida Dakterzada and colleagues [94] compare the value of various biomarkers in cerebrospinal fluid and plasma and demonstrate the superiority of central biomarkers over peripheral ones. Ashley F. Curtis et al. [95] explore the effects of American elderberry (*Sambucus nigra* subsp. *canadensis*) juice on cognition and inflammatory markers in patients with MCI. Gabriella Testa and colleagues [96] conducted an in-depth analysis of the possible role of proprotein convertase subtilisin/kexin type 9 (PCSK9) in the pathogenesis of AD, and its potential involvement in inflammation, oxidative stress, and Aβ deposition. Mădălina Georgeta Sighencea et al. [97] provide an interesting overview of the pathogenesis of the disease, including Aβ and tau pathology, glymphatic and lymphatic pathways, microbiota and the gut–brain axis, serotonergic and autophagy alterations, vascular dysfunction, the metal hypothesis, the olfactory pathway, and oral health; additionally, they explore potential molecular targets and novel promising approaches such as nanoparticle-based therapy, neural stem cell transplantation, vaccines, and CRISPR-Cas9-mediated genome editing techniques. David Vicente-Zurdo and colleagues [98] review the antioxidant potential of polyphenols for prophylactic and therapeutic purposes in AD.

With a certain critical perspective, we could say that the last 5 years of research have been relatively fruitful through providing better understanding of AD pathogenesis, the development of new predictive and diagnostic biomarkers, and the introduction of new therapeutic options for the treatment of AD, within the modest scope and limitations of immunotherapy.
